# Prenatal predictors of infant self-regulation: the contributions of placental DNA methylation of *NR3C1* and neuroendocrine activity

**DOI:** 10.3389/fnbeh.2015.00130

**Published:** 2015-05-29

**Authors:** Elisabeth Conradt, Mary Fei, Linda LaGasse, Edward Tronick, Dylan Guerin, Daniel Gorman, Carmen J. Marsit, Barry M. Lester

**Affiliations:** ^1^Department of Psychology, The University of UtahSalt Lake City, UT, USA; ^2^The Brown Center for the Study of Children at RiskProvidence, RI, USA; ^3^Department of Psychiatry, Warren Alpert Medical School of Brown UniversityProvidence, RI, USA; ^4^Department of Pediatrics, Warren Alpert Medical School of Brown UniversityProvidence, RI, USA; ^5^Department of Psychology, University of MassachusettsBoston, MA, USA; ^6^Department of Pharmacology and Toxicology and of Community and Family Medicine, Section of Biostatistics and Epidemiology, Geisel School of Medicine at DartmouthHanover, NH, USA

**Keywords:** DNA methylation, prenatal origins, glucocorticoid receptor gene, self-regulation, infancy

## Abstract

We examined whether placental DNA methylation of the glucocorticoid receptor gene, NR3C1 was associated with self-regulation and neuroendocrine responses to a social stressor in infancy. Placenta samples were obtained at birth and mothers and their infants (*n* = 128) participated in the still-face paradigm when infants were 5 months old. Infant self-regulation following the still-face episode was coded and pre-stress cortisol and cortisol reactivity was assessed in response to the still-face paradigm. A factor analysis of NR3C1 CpG sites revealed two factors: one for CpG sites 1–4 and the other for sites 5–13. DNA methylation of the factor comprising NR3C1 CpG sites 5–13 was related to greater cortisol reactivity and infant self-regulation, but cortisol reactivity was not associated with infant self-regulation. The results reveal that prenatal epigenetic processes may explain part of the development of infant self-regulation.

An infant’s ability to cope with stress is an important developmental achievement during the first year of life, and lays the foundation for later more complex forms of self-regulation. Following Rothbart (Rothbart et al., 2004), we view self-regulation as individual differences in one’s capacity to modulate reactivity. Difficulties in regulating in response to stress in infancy can set up a cascade of events leading to increased externalizing and internalizing behavior in early childhood (Moore et al., 2001; Olson et al., 2002). Given the importance of this milestone for later psychological health, there remains an unmet need to understand whether we can anticipate individual differences in self-regulation before the infant is even born.

A growing literature suggests that the origins of self-regulation in infancy may be identified prenatally (Van den Bergh et al., 2005; Glover et al., 2010; O’Donnell et al., 2013). Most of these studies focus on how prenatal stress, or exposure to prenatal psychopathology is predictive of infant temperament or problem behavior in childhood. For instance, greater stress exposure during pregnancy was related to poorer attention regulation at 8 months and more infant difficult behavior at 3 months (Huizink et al., 2003), as well as lower levels of disruptive temperament but also more problem and externalizing behavior at age two (Gutteling et al., 2005). Pregnancy-specific anxiety was also associated with lower mental and motor development at 8 months (Buitelaar et al., 2003). These findings suggest that certain temperamental and behavioral characteristics may have antecedents during the prenatal period.

What is unclear at this point is how prenatal exposures may be related to individual differences in infant self-regulation. While the processes are undoubtably complex, one likely candidate involves epigenetic mechanisms. Epigenetics is defined as inheritance of information based on gene expression control rather than on gene sequence (Berger et al., 2009). The most widely studied epigenetic mechanism tested in studies of human behavior is DNA methylation. DNA methylation is the process by which a methyl group is added to individual cytosines in the context of CpG dinucleotides. When this addition occurs in gene promoters, it is most often associated with transcriptional gene silencing, or the reduction of gene activity. Only one study that we know of includes prenatal epigenetic processes related to temperament in infancy. Alisch et al. (2014) using a rhesus macaque model, found that greater DNA methylation of *BCL11A* and *JAG1*, genes implicated in neurogenesis, were related to higher levels of anxious temperament in rhesus macaques.

It is likely that other genes related to the HPA response to stress may be involved. Chief among these may be the glucocorticoid receptor gene, or *NR3C1*. In humans, cortisol present in the bloodstream binds to glucocorticoid receptors, thereby reducing HPA axis activity and the release of more cortisol. DNA methylation reduces gene activity, and some have equated it to the “silencing” of a particular gene. Therefore DNA methylation of *NR3C1* should result in fewer glucocorticoid receptors to which cortisol can bind and therefore greater levels of cortisol in the blood, and possibly elevated resting cortisol and greater cortisol reactivity. Indeed, methylation of *NR3C1* has been associated with greater cortisol reactivity in 3 month-old infants exposed to prenatal maternal depression (Oberlander et al., 2008), and adults with a history of abuse in childhood (Tyrka et al., 2012). Greater cortisol reactivity in response to stress may in turn be associated with poorer self-regulation (Keenan et al., 2003), particularly if the stressor is outside of the infant’s control (Stansbury and Gunnar, 1994).

## Present Study

Our goal was to examine whether DNA methylation of *NR3C1* at birth explained individual differences in self-regulation in response to social stress at 4 months. We also questioned whether cortisol reactivity may explain how DNA methylation of *NR3C1* relates to infant self-regulation. We hypothesized that greater DNA methylation of *NR3C1* would be related to more cortisol reactivity and in turn more infant self-regulation.

## Method

### Participants

Mothers were recruited at birth from a local hospital following approval from the Women and Infants Hospital of Rhode Island and Dartmouth College IRBs. Only singleton, full-term (>= 37 weeks GA) infants were included in the study. Other exclusion criteria were maternal age <18 years or a life-threatening medical complication of the mother, congenital or chromosomal abnormality of the infant. When infants were 4 months old, mothers were invited into the laboratory for a face-to-face play assessment. Most of the participants were Caucasian (72.7%), with 12.5% African American, 3.1% Hispanic, 1.6% Asian, 0.8% American Indian, and 9.3% identifying themselves as “other”. Mother’s mean age was 30.5 years (range = 18–40 years). The sample included 128 infants (64 female) with an average age of 19.1 weeks (range = 13–26 weeks).

### Measures

#### DNA Methylation of NR3C1 at Birth

We sought to interrogate the 13 CpG sites in the *NR3C1* exon 1*_F_* promoter region. DNA from placenta samples (1 μg) were bisulfite-modified using the EZ DNA methylation Kit (Zymo Research) following the manufacturer’s protocol. Pyrosequencing was performed on PCR product amplified from bisulfite modified DNA. The primers for amplification were Forward: 5′-TTTTTTTTTT GAAGTTTTTT TA-3′ and Reverse:

5′-Biotin-CCCCCAACTC CCCAAAAA-3′. The first sequencing primer was designed to sequence the first five CpG sites (5′-GAGTGGGTTT GGAGT-3′), and the second sequencing primer was designed to sequence the following eight CpG sites (5′-AGAAAAGAAT TGGAGAAATT-3′) for a total of 13 sites sequenced. Percent DNA methylation at each CpG site was quantified using the Pyro Q-CpG software, version 1.0.11 (Qiagen). Bisulfite conversion controls were included on each sequencing read. In order for the sample’s DNA methylation extent to be called, the bisulfite conversion rate must be >93%, and for all samples examined the conversion rate was >95%. All samples were sequenced in triplicates from the same bisulfite converted DNA template, and if the repeats differed by >10% the sample was repeated.

### Infant Self-regulation

#### Face-to-Face Still-Face (FFSF) Assessment

Infant behaviors were coded during the double-exposure modification of Tronick’s FFSF (Haley and Stansbury, 2003). The assessment consisted of a two-minute unstructured play interaction, a two-minute still-face episode: a perturbation during which the mother is instructed to keep a still (“poker”) face and to look at the infant but not smile, talk, or touch the infant, a two-minute reunion episode which consists of an unstructured interaction during which the mother is asked to resume her normal play interaction with the infant and again is free to play, talk and touch the infant. In the double-exposure version of the FFSF, a second still-face and reunion episode are added. Because of our interest in self-regulation in response to stress, we only examined infant behaviors during the two reunion episodes.

The modified FFSF took place in an observation room equipped with an infant high chair. The observation room also included a swivel stool for the mother (with adjustable height), two cameras (one focused on the infant’s face and upper torso, the other on the mother’s face and upper torso). The signals from the two cameras was transmitted through a digital timer and split-screen generator into a video recorder to produce a single image with a simultaneous frontal view of the adult’s face, hands, and torso and the infant’s entire body.

The videos were coded using a modified version of the COPE method (COPE = comforting, object orientation, parent orientation, and escape; Braungart-Rieker et al., 1998). Infant self-regulation was comprised as a factor obtained using Principle Components Analysis that included: (1) any form of self-stimulation such as thumb/finger sucking, rubbing face/head/legs, rubbing seatbelt straps, and wringing hands (*M*_reunion 1_ = 0.69, *SD*_reunion 1_ = 0.48, *M*_reunion 2_ = 0.59, *SD*_reunion 2_ = 0.47), (2) and whether the infant looked away from his/her mother, which is seen as regulatory strategy involving attention (*M*_reunion 1_ = 0.55, *SD*_reunion 1_ = 0.23, *M*_reunion 2_ = 0.50, *SD*_reunion 2_ = 0.23; Manian and Bornstein, 2009). These behaviors were coded as 1 if it was present and 0 if it was absent during each 5-second interval of the reunion episodes. The factor analysis explained more than 55.45% of the variance and all factor loadings were above 0.57. Two coders were trained to code the infant videos and were reliable with each other at the end of 1 month. The intra-class correlation was 0.73 for self-stimulation and 0.93 for look-away.

#### Cortisol

Because of the diurnal rhythm of cortisol, all assessments took place in the morning between 8:00–11:30 AM (range = 8:11 AM 11:20 AM). Pre-stress cortisol samples were taken from infants upon entry into the laboratory, after informed consent was obtained. Post-stress cortisol was taken following the still-face paradigm (Tronick et al., 1978). Following Haley and Stansbury (2003), the post-stress saliva was taken 30 min after the end of the first still-face episode. Salivary cortisol was collected from the infant using a small sponge that was swabbed in the infant’s mouth until it became saturated with saliva. The swab was then placed into a storage vial and frozen until analyzed. Samples were analyzed by Salimetrics (Arizona) for analysis. If infants ate or drank 30 min prior to sample collection their mouths were swabbed with a wet paper towel.

### Missing Data

There were 149 infants with complete *NR3C1* methylation and self-regulation data. Of these, 9 children had missing *NR3C1* methylation data due to insufficient saliva volume needed for testing, 6 had missing cortisol data because the quantity of saliva was insufficient (*n* = 5) or because their cortisol values were extreme outliers (*n* = 1), yielding a final sample size of 128. Tests for birth and demographic differences between infants with and without missing data revealed that there were no differences in birth weight, gestational age, ethnicity, education level, or maternal age among infants with and without missing *NR3C1* methylation data (all *p*’s > 0.15) or missing cortisol data (all *p*’s > 0.10).

### Preliminary Analyses

Data were examined for outliers and violations of normality. For both the methylation and cortisol data outliers above or below 3 standard deviations were winsorized by replacing the value with the value at 3 standard deviations (<1% of values were affected). There was less methylation at the lower CpG sites (e.g., sites 1–4) compared to the later CpG sites. We therefore conducted a factor analysis to minimize the number of comparisons. The factor analysis revealed 2 factors explaining 51.96% of the variance: A factor that comprised CpG sites 1–4, and a factor for sites 5–13. All factor loadings were above 0.52. There was still a slight positive skewness in the factors so we report spearman rank correlations when analyzing methylation data. The raw cortisol values (μg/dL) were positively skewed and normalized using a log transformation.

## Results

### Covariates

We examined the time of each cortisol assessment relative to each measure of cortisol (e.g., whether time of the pre-stress measurement was correlated with the pre-stress cortisol value). Time of measurement was not significantly related to the time-specific measurement of cortisol (all *p*’s > 0.35). We also examined whether either infant or maternal prescription and/or non-prescription steroid medication, or maternal use of caffeine impacted cortisol concentrations. Steroid use within the last twelve hours by either mother or infant was not significantly associated with the cortisol values (all *p*’s > 0.40), and neither was maternal consumption of caffeine that morning (*p*’s > 0.11). If infants had eaten less than 30 min prior to cortisol sampling their mouths were rinsed with water. As nap times may also affect cortisol values we examined whether time of nap and/or time of awakening affected cortisol. Neither was related to our cortisol values (*p*’s > 0.18).

We also examined covariates that may be related to DNA methylation of *NR3C1*, cortisol, or self-regulation in response to the still-face episode. These covariates include birth weight, gestational age, ethnicity, and sex. None of these covariates were significant predictors of DNA methylation of *NR3C1*, cortisol, or self-regulation (all *p*’s > 0.08).

### Is DNA Methylation of NR3C1 Related to Neuroendocrine Functioning?

**Figure 1:**
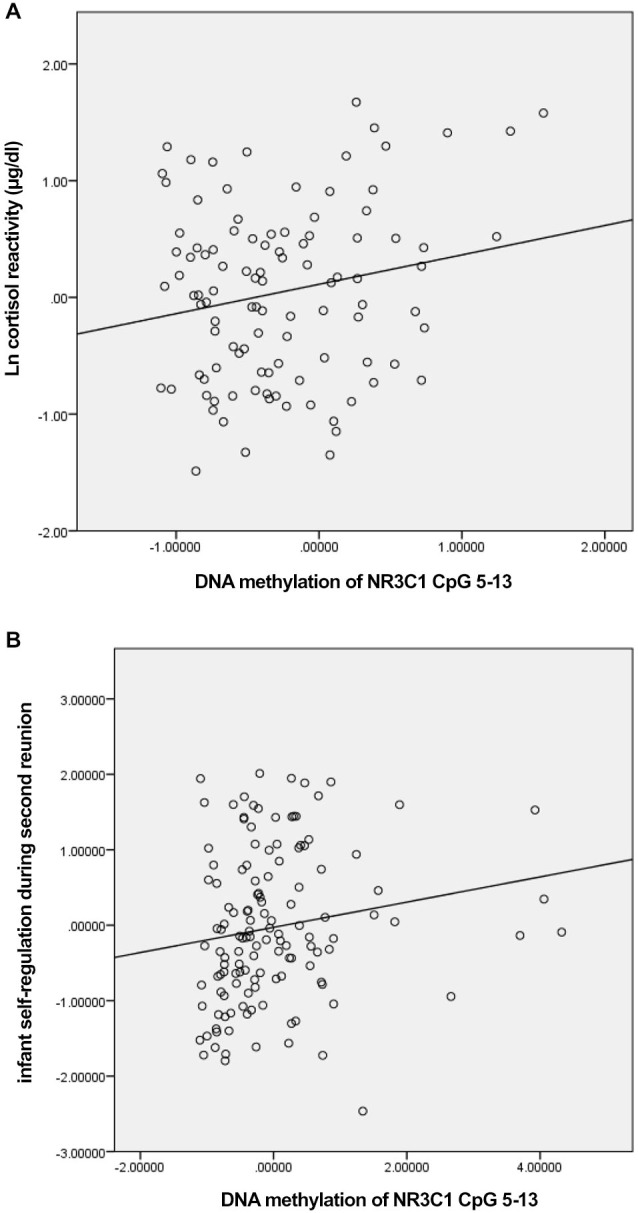
**Scatterplots for the correlation between DNA methylation of *NR3C1* CpG 5–13 factor score and cortisol reactivity (A) and infant self-regulation following the second still-face episode (B)**.

We first tested whether DNA methylation of *NR3C1* was related to pre-stress cortisol and cortisol reactivity in response to the still-face paradigm (Table 1). DNA methylation of the factor comprising *NR3C1* CpG sites 5–13 was related to greater cortisol reactivity, *ρ* = 0.19, *p* < 0.05 (Figure 1A). There were no other significant associations.

**Table 1:** **Means, standard deviations, and correlations of variables of interest**.

Variable	M	SD	1	2	3	4	5
1. NR3C1 factor 1 (CpG sites 1–4)	0	1.00	–				
2. NR3C1 factor 2 (CpG sites 5–13)	0	1.00	0.12	–			
3. Ln Pre-stress cortisol (μg/dl)	−1.79	0.67	−0.18	−0.06	–		
4.Ln cortisol reactivity (μg/dl)	1.30	1.81	0.19	0.19*	−0.64***	–	
5. self-regulation reunion1	0	1.00	−0.09	0.02	−0.14	−0.11	–
6. self-regulation reunion2	0	1.00	−0.10	0.25**	−0.04	0.06	0.01

Note: *p < 0.05, **p < 0.01, ***p < 0.001.

### Is DNA Methylation of NR3C1 Related to Self-Regulation in Response to Stress?

We then examined associations between the DNA methylation factors and infant self-regulation; specifically, we examined associations between: DNA methylation of *NR3C1* CpG sites 1–4, DNA methylation of *NR3C1* CpG sites 5–13, infant self-regulation following the first still-face episode, and infant self-regulation in response to the second still-face episode (Table 1). DNA methylation of *NR3C1* CpG sites 5–13 was related to greater self-regulation following the second still-face episode, *ρ* = 0.25, *p* = 0.004 (Figure 1B). None of the other associations were significant.

### Is Neuroendocrine Functioning Related to Infant Self-Regulation in Response to Stress?

Finally, we examined whether cortisol reactivity was related to infant self-regulation in response to the first and second still-face episodes. There were no effects between pre-stress cortisol, cortisol reactivity or infant self-regulation.

## Discussion

Our goal was to examine prenatal epigenetic predictors of infant self-regulation and processes that may explain how these epigenetic predictors relate to self-regulatory behaviors at 4 months. We found that greater DNA methylation of CpG sites 5–13 on *NR3C1*, involved in the neuroendocrine response to stress, was predictive of more cortisol reactivity and infant self-regulation in response to social stress. However, cortisol reactivity was not related to infant self-regulation.

There is growing interest in translational work aimed at understanding whether DNA methylation may be a process by which prenatal exposures impact individual differences in neurodevelopment. A number of studies using animal models suggest that prenatal stress (Mueller and Bale, 2008) and the quality of the early rearing environment (Liu et al., 1997) are related to DNA methylation of genes involved in the stress response, and in turn HPA axis functioning. Oberlander and colleagues (Oberlander et al., 2008) were the first to find that greater methylation of *NR3C1* at CpG site 3 in cord blood was predictive of greater cortisol reactivity in 3-month infants. Tyrka and colleagues (Tyrka et al., 2012) also focused on exon 1*_F_* of *NR3C1* in adult humans with a history of childhood abuse and found that more mean methylation of sites 7–13 were related to less cortisol reactivity, which is consistent with the theory of allostatic load that wear and tear on the neuroendocrine system would be related to attenuated cortisol responses to stress over time. Our methylation and cortisol reactivity findings were specific to sites 5–13, and not 1–4. CpG sites 7 and 12 are binding sites for the transcription factor SP1 (Armstrong et al., 2014). SP1 is a mediator of nuclear signaling in response to hormones and therefore increased DNA methylation at these sites could decrease SP1 binding and subsequent transcription, which may ultimately interfere with HPA axis regulation. This process could explain why we found increased cortisol reactivity in CpG sites implicated in SP1 binding. However, we did not directly interrogate SP1 activity and at this point this hypothesized process is purely speculative.

DNA methylation of *NR3C1* CpG sites 5–13 was also related to infant self-regulation at 4 months. There is very little research linking DNA methylation to infant behavior. Previous work in newborns shows that DNA methylation of *NR3C1* is related to lethargy, self-regulation, hypotonia, quality of movement, and attention (Bromer et al., 2013; Conradt et al., 2013). It may be that, with development, the newborns with these poor neurobehavioral profiles require more attempts at self-regulation in response to stress at 4 months. Though preliminary and in need of replication, our findings highlight the utility of using DNA methylation of *NR3C1* in predicting infant self-regulation 4 months later.

Cortisol reactivity did not mediate the effect of DNA methylation on infant self-regulation. The development of self-regulation is undoubtedly complex and is likely accounted for by DNA methylation of a number of different genes in addition to *NR3C1*. For instance, DNA methylation of *11β-HSD2*, a gene involved in converting cortisol to inert cortisone, has been implicated in newborn neurobehavior, including quality of movement (Marsit et al., 2012), and hypotonia (Conradt et al., 2013). It is also likely that more proximal variables, such as variation in parental behavior, exert a stronger influence on the development of infant self-regulation than cortisol reactivity (Haley and Stansbury, 2003). In addition, other physiological systems, such as the autonomic nervous system, may be a stronger predictor of infant self-regulation than the neuroendocrine response (Haley and Stansbury, 2003; Conradt and Ablow, 2010), particularly since the autonomic system is activated immediately following stress while the time course of the neuroendocrine response to stress is longer (e.g., typically 20–30 min following the stress exposure).

As some of the original rodent work was conducted among rats reared in extremely high vs. low quality caregiving environments, it may be that we will find effects emerge in environments more extreme for high vs. low early life stress or socio-economic status. Thus, a logical extension of this work is to examine relations between DNA methylation, cortisol reactivity, and infant stress responses in cohorts exposed to high vs. low early life stress. This kind of analysis would enable us to extend this research by examining placental DNA methylation as a biomarker of problem behavior, such as externalizing or internalizing behavior, later in life. We also only examined DNA methylation in one gene. Bioinformatic approaches identifying gene networks involved in the development of infant regulatory responses are needed as there is clearly not one gene responsible for the development of infant self-regulation.

While it is generally accepted that the behaviors we observed are self-regulatory in nature, we cannot rule out the alternative explanation that these behaviors may also reflect signs of stress. While these behaviors were observed in what may putatively be a less-stressful context (face-to-face play with the mother following the still-face), and therefore regulatory in nature, it is possible that the coded behaviors (e.g., thumb sucking, touching high chair straps) reflect a carry-over of feelings of stress from the still-face episode. Indeed both may be true and future research might utilize psychophysiologic measures to help with this distinction.

We identified relations between DNA methylation of *NR3C1* at birth, cortisol reactivity, and infant self-regulation at 4 months. If individual differences in DNA methylation of genes involved in the infant stress response are to be used as biomarkers for adverse social and emotional outcomes in infancy it is imperative that large-scale, longitudinal data incorporating DNA methylation from a variety of genes are collected to map pathways leading to problem behavior in early childhood, beginning at birth. We hope that these results stimulate similar research in this area, with the long-term goal of fostering health social and emotional development.

## Conflict of Interest Statement

The authors declare that the research was conducted in the absence of any commercial or financial relationships that could be construed as a potential conflict of interest.
